# A proposed approach to defining per- and polyfluoroalkyl substances (PFAS) based on molecular structure and formula

**DOI:** 10.1002/ieam.4735

**Published:** 2023-02-10

**Authors:** Linda G. T. Gaines, Gabriel Sinclair, Antony J. Williams

**Affiliations:** 1 Office of Superfund Remediation and Technology Innovation, Office of Land and Emergency Management, US Environmental Protection Agency, DC, Washington, USA; 2 ORAU Student Services Contractor to Center for Computational Toxicology and Exposure, Office of Research and Development, US Environmental Protection Agency, NC, Research Triangle Park, USA; 3 Office of Research & Development, Center for Computational Toxicology & Exposure, US Environmental Protection Agency, NC, Research Triangle Park, USA

**Keywords:** Cheminformatics, Environmental chemistry, Per- and polyfluoroalkyl substances, PFAS

## Abstract

Various groups and researchers, including the authors of this work, have proposed different definitions of what constitutes per- and polyfluoroalkyl substances (PFAS). The different definitions are all based on a structural definition. Although a structural definition is reasonable, such an approach is difficult to execute if the intent is to narrow or refine the definition. This approach can also lead to inexplicable demarcations of what are and what are not PFAS. Our objective was to create a narrow, simple PFAS definition that allows interested groups to communicate with a common understanding and will also serve as a starting point to focus on substances that are of real environmental concern. Our studies have demonstrated that numerous highly fluorinated complex structures exist that make a structure-based definition difficult. We suggest that a definition based on fractional fluorination expressed as the percentage of fluorine of a molecular formula using atom counting offers advantages. Using a combination of a structure-based definition and a definition based on the fractional percentage of the molecular formula that is fluorine can provide a more inclusive and succinct definition. Thus, we propose a new definition based on four substructures along with any structures where the molecular formula is 30% fluorine by atom count. *Integr Environ Assess Manag* 2023;00:1–15. Published 2023. This article is a U.S. Government work and is in the public domain in the USA. *Integrated Environmental Assessment and Management* published by Wiley Periodicals LLC on behalf of Society of Environmental Toxicology & Chemistry (SETAC).

## BACKGROUND

Justice Potter Stewart, in concurring on the decision in *Jacobellis v. Ohio*, 378 US 184, stated “I shall not today attempt further to define the kinds of material I understand to be embraced within that shorthand description; and perhaps I could never succeed in intelligibly doing so. But I know it when I see it, and the motion picture involved in this case is not that” (“Jacobellis v. Ohio,” 1963). Most readers will likely recognize this phrase as representative of the judge’s position on the recognition of adult material under review, summarily reduced to “I know it when I see it.” Proposing a definition of per- and polyfluoroalkyl substances (PFAS) provides a similar dilemma, especially if attempting to provide a succinct definition.

**FIGURE 1:**
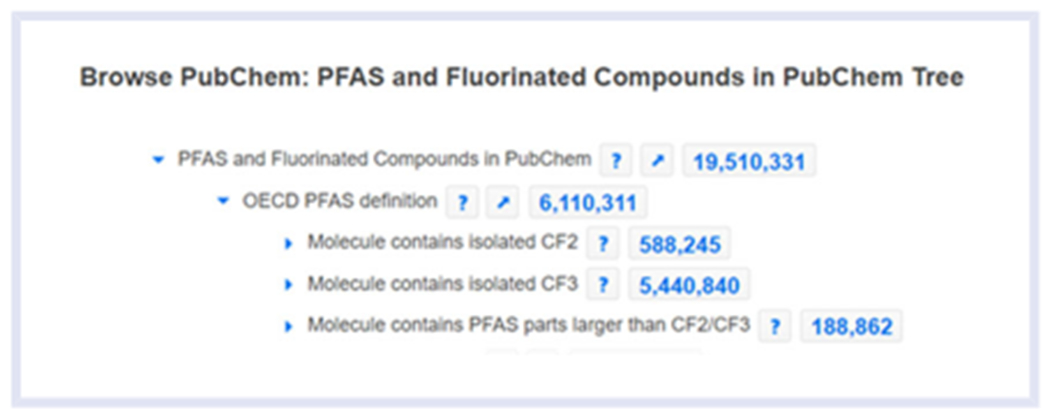
A snapshot of a *fraction* of the PubChem classification browser per- and polyfluoroalkyl substances (PFAS) tree as of 10 July 2022

Buck et al. (2011) first defined PFAS as aliphatic substances that “contain 1 or more carbon atoms on which all of the hydrogen substituents (present in the nonfluorinated analogs from which they are notionally derived) have been replaced by fluorine atoms, in such a manner that they contain the perfluoroalkyl moiety (–C_n_F_2n+1_–).” Ten years later, the Organisation for Economic Co-operation and Development (OECD) proposed a revised, even more general, but easily implementable PFAS definition of “fluorinated substances that contain at least one fully fluorinated methyl or methylene carbon atom (without any H/Cl/Br/I atom attached to it).” This interpretation means that “with a few noted exceptions, any chemical with at least a perfluorinated methyl group (–CF_3_) or a perfluorinated methylene group (–CF_2_–) is a PFAS.” This revised definition (hereafter referred to as OECD2021) removes the requirement that the structure is entirely aliphatic, and only requires that the minimal fully fluorinated methyl or methylene group is saturated and aliphatic (OECD, 2021). OECD2021 includes approximately 38 000 structures currently in the USEPA’s public version of the CompTox Chemicals Dashboard (Williams et al., 2022; from here on the Dashboard) and more than 6.1 million structures in PubChem (as of July 2022; Figure 1; National Library of Medicine, 2022). These databases continue to expand as new content is registered, and the number of structures fitting this simple definition is certain to increase.

These definitions are simple because they require minimal fluorination and a few other structural detail requirements. However, this simplistic approach can lead to complex issues by creating a huge group of chemicals with vastly different physicochemical, toxicity, and fate and transport properties as well as uses. Narrowing the definition to delineate a more manageable set of chemicals is necessary to focus on regulation and research needs, for example, but a structure-based definition may or may not allow focusing on PFAS with similar properties. For example, in 2018, OECD published a “Global Database of Per- and Polyfluorinated Substances” that focused on chemicals with a perfluoroalkyl moiety of three or more carbons (i.e., –C*_n_*F_2*n*_–, *n* ≥ 3) or a perfluoroalkylether moiety of two or more carbons (i.e., –C*_n_*F_2*n*_OC*_m_*F_2*m*_–, *n* and *m* ≥ 1; OECD, 2018). However, the authors are not aware of the discussions that led to this particular definition although OECD states that the study builds on efforts to identify PFAS that may have been on the market using a carefully designed methodology (OECD, 2018). This study, however, provides a more focused database significantly reducing the number of PFAS included to approximately 6000 structures in the Dashboard (Williams et al., 2022). The OECD used the more focused list to identify PFAS structures that had not been identified before, determine those that were commercially available, and identify new groups of PFAS (OECD, 2018).

In June 2021, USEPA’s Office of Pollution Prevention and Toxics proposed a different definition of PFAS for proposed reporting and recordkeeping requirements under the Toxic Substances Control Act (TSCA), which would require manufacturers and importers of the described chemicals to report uses, production volumes, disposal, exposures, and hazards. For that specific proposed rule, PFAS were defined as any chemical substance or mixture that included the structural unit R–(CF_2_)–C(F)(R’)R”. Both the CF_2_ and CF moieties are saturated carbons, but none of the R groups (R, R’, or R”) can be hydrogen (hereafter referred to as TSCA2021; USEPA, 2021c). Although not included in the proposed rule, USEPA’s Office of Chemical Safety and Pollution Prevention stated in a letter to Robert Sussman responding, on reconsideration, to a petition it received under section 21 of the TSCA from the Center for Environmental Health, Cape Fear River Watch, Clean Cape Fear, Democracy Green, Toxic Free NC, and the NC Black Alliance (petitioners) on 14 October 2020 that the working definition “is focused on substances likely to be present in the environment, especially water, thereby focusing on substances with greater potential for exposures to people/ environment and by extension more potential to present risks. For example, chemicals with (–CF_2_–) that are not (–CF_3_) are expected to degrade in the environment, and most substances with only one terminal carbon (–CF_3_) are expected to degrade to trifluoroacetic acid, which is a well-studied substance. This working definition focuses on PFAS believed to be of highest concern based on their persistence and potential for presence in the environment and human exposure” (USEPA, 2021b).

Conversely, in the nonscientific media and literature, PFAS have been referred to as “highly fluorinated.” This phrase is simple, understandable, and logical, as a main concern with PFAS is their resistance to degradation due to stability of the carbon–fluorine bond. Thus, the more highly fluorinated the organic molecule is, the more resistant to degradation the molecule is (Zhang et al., 2022). However, what degree of fluorination (i.e., how many fluorine atoms are associated with a chemical structure) defines a highly fluorinated compound? Further, how can a qualitative term like highly fluorinated be translated into a usable definition? A substance meeting the basic requirement of OECD2021 in having only one fully fluorinated carbon cannot be described as highly fluorinated with a few minor exceptions. The more focused lists of TSCA2021 and OECD2018 move closer to the “highly fluorinated” description.

The question of how much fluorination is highly fluorinated, at least to some extent, is important for the intended use of a particular PFAS definition. Previously, we reviewed various PFAS definitions, including those mentioned above, and what compounds would be considered a PFAS using them (Williams et al., 2022). The differences in the various PFAS definitions, as well as what structures would be encompassed, is important to ensure it is appropriate for intended applications. Similarly, a review of the different definitions demonstrates the large variation in the numbers of chemicals that would be considered as PFAS, based on the different definitions, including the variations in chemical fluorination (Williams et al., 2022).

The DSSTox database (Grulke et al., 2019) contains all chemicals underpinning the Dashboard (Lowe & Williams, 2021; Williams et al., 2017), and a search of the publicly accessible version of the database (~906k chemicals as of July 2022) indicates that the number of structures that fit a particular PFAS definition vary from approximately 6000 structures for OECD2018 to 38 000 structures for OECD2021 (Williams et al., 2022). The results from the Dashboard search contrast with the much longer list of chemicals available on PubChem, and specifically under their PFAS Classification Browser (https://pubchem.ncbi.nlm.nih.gov/classification/#hid=120), a classification tree to browse, and other fluorinated compounds present in PubChem. In the classification tree, the “OECD PFAS definition” node matches the OECD PFAS definition of saturated CF_2_, and as of July 2022 and as shown in Figure 1, more than 6 million molecules match OECD2021 with 5.4 million having an isolated CF_3_ and ~600 k with an isolated CF_2_ (REF: https://gitlab.lcsb.uni.lu/eci/pubchem-docs/-/raw/main/pfas-tree/PFAS_Tree.pdf). Thus, one of the world’s most popular chemical-related websites demonstrates the vast and expansive listing of compounds captured under OECD2021.

In related work, Barnabas et al. (2022) reported on their work searching open access data including CORE (Connecting REpositories; Open University and Jisc, 2022) and Google Patents (Google, 2022). In their work, they used three specific PFAS classes based mainly on others’ definitions (with associated download files at https://zenodo.org/record/6474542#.YssIFnbMI2w; Barnabas et al., 2022). These classes and the total number of structures found are summarized in Supporting Information: Table S1. Their work introduces yet another definition and demonstrates the structures resulting from the three PFAS classes based on different databases.

The Dashboard has assembled numerous PFAS lists (https://comptox.epa.gov/dashboard/chemical-lists?filtered=&search=PFAS), including lists of those based on various definitions (Williams et al., 2022). One of these collections describes the versioned iterations of the PFAS Structures list (PFASSTRUCT: https://comptox.epa.gov/dashboard/chemical-lists/PFASSTRUCT), which is a list of structures in the Dashboard that fit a particular structural definition of PFAS. The first version (PFASSTRUCTv1) was assembled in 2018 and was described as structures fitting a filter description “designed to be simple, reproducible and transparent, yet general enough to encompass the largest set of structures having sufficient levels of fluorination to potentially impart PFAS-type properties.” Thus, the goal since the beginning of PFASSTRUCT has been to have easily understandable criteria that define the specifications for a PFAS. Since its inception, PFASSTRUCT has provided a more focused list of PFAS than is captured by other definitions such as that in Buck et al. (2011) or OECD2021, and, as a result, users can more efficiently and easily search PFASSTRUCT for structures of potential concern, specifically highly fluorinated structures. For example, PFASSTRUCTv1 served as a starting point for research efforts (Williams et al., 2022).

**FIGURE 2:**
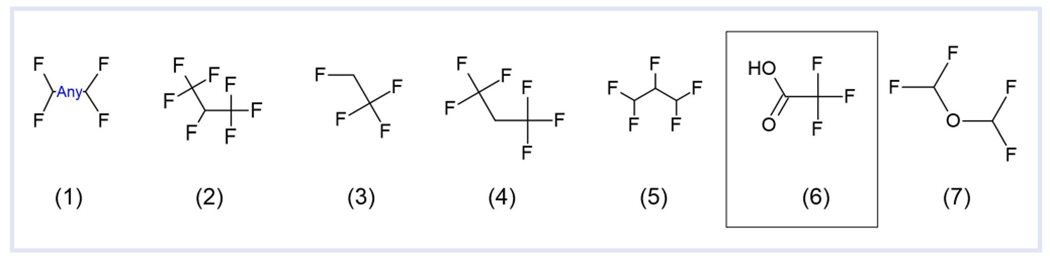
Substructures used for PFASSTRUCTv3 and PFASSTRUCTv4

The assembly of the different versions of PFASSTRUCT have been discussed previously (Williams et al., 2022) and are summarized in Table 1, with the substructures used for PFASSTRUCTv3 and PFASSTRUCTv4 provided in Figure 2. Each version supersedes and improves on the previous version, but all versions are still available. Importantly, the number of structures included in the different versions of PFASSTRUCT changes because of both the change in the description of what is included and based on the number of structures in the Dashboard. Of note, in the Dashboard, structures include multicomponent chemicals such as salts and mixtures. For example, trifluoroacetic acid (Substructure 6 of Figure 2) is found in many chemical solutions as it is used as a solvent and thus is a component of many structures.

While comparing the previously generated PFAS lists with the entirety of the DSSTox content, it was determined that certain structures that are inarguably PFAS, “we know them when we see them,” were not captured in PFASSTRUCTv4. For example, highly branched aliphatic structures consisting only of carbon and fluorine, and other examples listed in the Results section, were not in PFASSTRUCTv4 because they did not include one or more of the necessary substructures. Thus, it was realized that an analysis of PFAS structures that were on one list, such as OECD2021 or TSCA2021, but not on PFASSTRUCTv4 was required to determine which additional substructures would need to be added to the existing PFASSTRUCTv4 substructure set to capture all inarguably PFAS structures. The goal was to improve PFASSTRUCT to allow it to continue to be used as a starting point for research and other related efforts.

This article describes the process used to determine a theoretically better, descriptively succinct, and more focused definition for PFAS that was used to develop PFASSTRUCT version 5 (hereafter PFASSTRUCTv5). The goal was to capture all inarguably PFAS structures. The problems encountered in using a completely structure-based definition and a new approach to describe PFAS are also described.

## METHODS

### Substructure analysis

The PFAS lists previously generated for the Dashboard were reexamined focusing on the latest PFASSTRUCTv4. The process used for the analysis is displayed graphically in Supporting Information: Figure S1. PFASSTRUCTv4 contains structures that have one or more of the substructures shown in Figure 2, excluding Substructure 6, which was only used for PFASSTRUCTv3. Six lists were generated, with each including structures that include the respective substructures. The six lists that create PFASSTRUCTv4 were compared and analyzed using SAS version 9.4 (TS1M1; SAS Institute). The six lists were analyzed to determine overlap and thus which structures contain more than one substructure and which contain only one substructure. Thus, it was determined how many structures were unique to each list. This allowed for a determination of whether any of the substructures were extraneous.

PFASSTRUCTv4 (~11 000 structures) was further analyzed to determine what additional substructures were needed to capture all PFAS. The process used for the analysis is displayed graphically in Supporting Information: Figure S2. PFASSTRUCTv4 was compared with the list of structures containing at least one CF_2_, with no further requirements (38 000 structures). It had previously been noted that some structures fit TSCA2021 but were not on PFASSTRUCTv4, so TSCA2021 (9000 structures, available in Williams et al. [2022]) was also compared with these lists. A combined list containing structures from all these lists was generated. All structures with fewer than three fluorine atoms were then removed to allow for a more focused analysis. The Simplified Molecular Input Line Entry System (SMILES) of each structure was analyzed to determine whether there were one or more terminal CF_3_ groups. To further narrow the analysis, if the structure consisted of only three fluorine atoms in a CF_3_ group, the structure was not further analyzed. Similarly, if the structure contained only six fluorine atoms associated with two CF_3_ groups, the structure was not analyzed further if those CF_3_ groups were not connected to the same carbon because these structures would not fit many definitions of PFAS. The narrowed analysis was aimed at finding highly fluorinated structures that were not captured by PFASSTRUCTv4.

Additional analysis excluded any structures that included one of the PFASSTRUCTv4 substructures shown in Figure 2. The assumption was made that any structure that was already on PFASSTRUCTv4 should be considered a PFAS. Thus, removing those structures allowed for a focused analysis of structures that do not contain any Figure 2 substructures but might still be considered PFAS. Thus, this allowed for focusing on methods to improve PFASSTRUCTv4. The focused list of PFAS of approximately 3000 structures consisted of those with three or more fluorine atoms where the structure did not consist of solely one or two terminal CF_3_ groups and did not fit one of the PFASSTRUCTv4 substructures.

Visual analysis of the different structures was accomplished by creating an SDF file and reviewing in the ACD/Labs SDFViewer module included with ACD/ChemSketch software (Advanced Chemistry Development Inc.), which allows for reviewing molecular structures in either a tile or table view so that clusters of chemicals could be examined easily. The software also allows for substructure searching and an easy way to look at the resulting collection of hits. Various substructures were identified as PFAS, and those substructures were defined, so they could be used to search the DSSTox database. The resulting structures identified in the DSSTox database were then reviewed.

### Analyzing the percentage of fluorine

While reviewing the substructures needed to capture the structures of the PFAS, it was determined that this repetitive and iterative method of finding PFAS structures based solely on substructures may not be the best method, and we decided that a different approach might be necessary. The review process suggested that structures subjectively considered to be PFAS were composed of either 30% or 40% fluorine, based on the fraction of the molecular formula excluding hydrogen atoms. For example, for a compound with the molecular formula C_6_HF_9_O_6_, the percentage of fluorine excluding hydrogen contained in the formula would be 9F/(6C + 9F + 6O) = 42%. If hydrogen is included, the fluorine percentage would be 9F/(6C + 1H + 9F + 6O) = 40%. This formula is based strictly on atom counting without considering the weight of the atoms, which could change percentages significantly. For example, perfluorobutanoic acid (PFBA; DTXSID4059916), its sodium salt (DTXSID70880179), and its silver salt (DTXSID70880199) all have the same fluorine percentage based on the formula, which is 7F/(4C + 1(H/Na/Ag) + 7F + 2O) = 50%. Conversely, if atomic weights were considered, they would all have the same molecular weight of fluorine (7 × 19 = 133), but the molecular weight fluorine percentage of the different compounds would be 133/214 = 62% for PFBA, 133/236 = 56% for Na PFBA, and 133/321 = 41% for Ag PFBA. Thus, for the same amount of fluorine, the salts, in particular the silver salt, would seem significantly less fluorinated numerically than the acid.

The DSSTox database was then searched for structures based on the percentage of fluorine using the definition provided above (i.e., fraction based on the molecular formula and ignoring hydrogen atoms in the formula). The search results are presented in Table 2, and the process used for the analysis is displayed graphically in Supporting Information: Figure S3. The reported search results are all approximate. Comparison of these lists highlighted different structures to consider for inclusion.

### Substructures and percentage of fluorine

The proposed PFASSTRUCTv5 criteria were developed by testing combinations of the percentage of fluorine and substructure-based criteria. The resulting PFASSTRUCTv5 list was compared with PFASSTRUCTv4 and targeted PFAS lists to ensure that it captured all previously identified PFAS. During finalization of PFASSTRUCTv5, free radicals were removed from the list. The list was also filtered to remove any structures without a C–F bond as several mixtures of hydrocarbons in HF and similar substances were found via the search for the percentage of fluorine.

## RESULTS

### Substructural analysis

PFASSTRUCTv4 was analyzed both to determine whether it could be simplified by removing unneeded substructures and to identify whether additional substructures were needed to capture structures not detected previously. Initial analysis of PFASSTRUCTv4 substructures (Figure 2) revealed that Substructure 2 was extraneous. There were no structures unique to this list. Substructure 1 is indicated as “any” for the bond between the two carbons, but the only compound where the bond could be a double bond and still have two fluorine atoms attached to both carbons is tetrafluoroethene, which is not considered a PFAS by most definitions including OECD2021. Therefore, the substructure could be simplified by removing the “any” indicated. Substructure 4 allows for many branched structures, whereas Substructure 5 allows for certain structures with a hydrogen that are not in TSCA2021. Substructure 7 uniquely allows for ethers but no other heteroatoms.

Analysis of the lists resulting from a formula-based query revealed some structures with rather unique complex structures that were not included in many previous structure-based definitions. Table 3 presents examples of these structures. A particular substructure might be necessary for a structure to be included, but that substructure might only be needed for a very small number of structures. For example, tetrakis(trifluoromethoxy)methane, shown in Table 3, is currently the only structure in DSSTox to need the substructure CF_3_–O–C–O–CF_3_. Including additional substructures like CF_3_–C–C–CF_3_ will allow the capture of perfluorohexamethylprismane, but it will also capture 1,1,1,4,4,4-hexafluoro-2,3-diphenylbutane-2,3-diol. Although it can be argued whether or not this latter chemical is a PFAS, from an environmental perspective the two benzene rings may be of more concern in terms of toxicity and degradation than the two completely fluorinated terminal carbons.

Based on a comprehensive analysis of the structures, a list of 15 substructures was proposed to represent all related structures that should be included in PFASSTRUCTv5. Some of these substructures would be similar to the existing set (Figure 2). Some of the existing substructures and some of the new substructures would need an expanded list of noncarbon heteroatoms (e.g., CF_3_–X–C–X–CF_3_, where X can be N, O, or S) to allow for functional groups other than ethers such as sulfides and amines. Although this revised list of substructures would expand and improve the coverage of PFASSTRUCTv5 by including certain PFAS not included in PFASSTRUCTv4, the increasing number of substructures would make assembling the list more complex and hardly succinct and thus more difficult to describe. Further, as new structures were added to the DSSTox database, it was possible that the substructures list might require even more revision to capture increasingly complex structures.

### Percentage of fluorine analysis

As a result of the obvious complexity associated with attempting to assemble multiple complex substructures, a different approach was proposed as a path to search for compounds. This approach used the percentage of fluorine contained in the molecular formula rather than an increasingly complex list of substructures. It should be noted that OECD has stated that the term highly fluorinated cannot and should not be translated literally into the weight percentage of fluorine atoms in a molecule and notes that highly fluorinated is an ambiguous term. Three 6:2 fluorotelomer-based compounds (6:2 FTOH, DTXSID5044572; C_6_F_13_C_2_H_4_SO_2_ NHC_3_H_6_N(O)(CH_3_)_2_, DTXSID80880983; and 6:2 fluorotelomer ethoxylates [C6F13–(CH_2_CH_2_O)n–H, *n* = 0–13]) were used as examples by OECD, revealing the differing fluorine content between the examples (67.8, 46.7 wt%, and lower when *n* > 4, respectively), although they have the same perfluorohexane substructure (OECD, 2021). As described in the Methods section, the approach described herein is not a weight percentage but a formula count percentage, although it is similar in approach to that argued against by OECD. We believe it is part of a fitting approach to developing the next iteration of PFASSTRUCT.

PFASSTRUCTv4 was analyzed to determine what fluorine fraction of the molecular formula would intuitively be needed to define a PFAS. In general, this approach worked very well, and the use of fluorine percentage in the formula, excluding the contribution from hydrogen atoms, was significantly more inclusive of chemicals deemed to be PFAS, based on list comparison. As would be expected and was predicted by OECD, even this approach was not perfect, and a certain number of outliers were identified. Outliers included structures where the fluorinated portion was a relatively small part of the molecule, but with the fluorinated portion concentrated in one section of the molecule, that section would be identified as PFAS, and thus the entire structure would be identified as PFAS because the structure would degrade to a PFAS. If a substance degrades to a PFAS, then that original substance would be considered both a PFAS and a PFAS precursor. Examples of these types of structures are provided in Table 4. All examples in Table 4 are in PFASSTRUCTv4.

Another consideration with this approach is what fluorine percentage is appropriate to represent a PFAS molecule. This question is clearly subjective. Initial analysis of a few of the more complex structures that did not fit the substructures used in PFASSTRUCTv4 indicated that they contained at least 40% fluorine in the molecular formula, when hydrogen atoms were excluded. To determine what percentage was most appropriate, structures with lower fluorine percentage were examined, and initial analysis of the existing chemicals in the DSSTox database indicated between 20% and 40% is probably appropriate, based on what percentage was needed to capture structures that meet different structure-based PFAS definitions. A large gray area exists with example structures given in Supporting Information: Table S2.

### Substructures and percentage of fluorine

**FIGURE 3:**
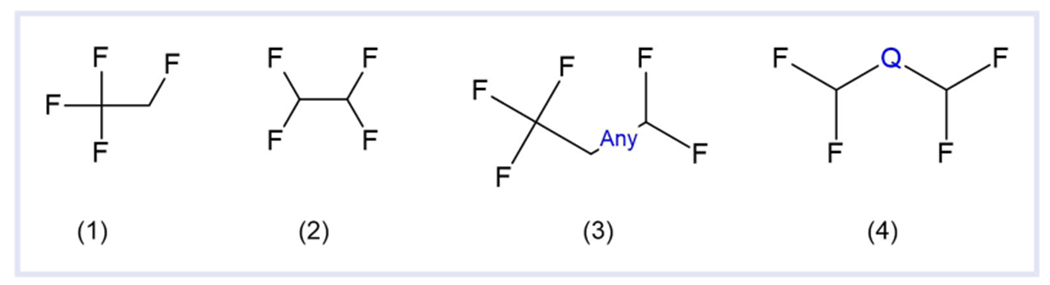
Substructures used in combination with percentage of fluorine for PFASSTRUCTv5

Having demonstrated that neither substructures nor the percentage of fluorine in the formula could provide the desired results alone in terms of a one-size-fits-all solution to defining PFAS chemicals, according to our expectations of “we know it when we see it,” a combined approach was deemed necessary. The use of the 30% fluorine without hydrogen would allow for inclusion of some of the complex highly fluorinated structures. Without the use of a percentage of fluorine count, these structures would require a multitude of substructures to include in a definition. Hence, by including structures identified using a percentage of fluorine, the substructure search could be simplified significantly, one of the objectives for this revised definition. Conversely, the use of a few basic substructures would ensure that certain structures would not be missed when considering the size of the structure and the small fractional contribution of the fluorine count. This would address the appropriate concerns that were noted by the OECD. Four substructures were determined to be required in combination with the fractional percentage of fluorine. These structures are simple and are shown in Figure 3. For Substructure 4 the heteroatom Q can be B, O, N, P, S, or Si.

This formula percentage of fluorine part of the definition captures all the unique structures given as examples in Table 3 with the exception of 1,1,1,4,4,4-hexafluoro-2,3-diphenylbutane-2,3-diol (DTXSID40313106). 1,1,1,4,4,4-Hexafluoro-2,3-diphenylbutane-2,3-diol does not fit the substructures in Figure 3 and is only 25% fluorine. Conversely, the substructure portion of the definition captures all the examples provided in Table 4. However, this definition includes some structures that other definitions may exclude. Examples are provided in Table 5.

Several of these chemicals are certainly outside the bounding term PFAS that limits interpretation to alkyl substances, but the dominance of fluorine as a fractional percentage of the molecular formula is obvious. This expanded definition would also include other structures that most would agree are not PFAS in terms of lacking any fully fluorinated alkyl groups. A restriction could be placed on the search such as minimum carbon or fluorine, but that would increase the complexity of the definition. Another restriction that could be added would be to require an alkyl bond with respect to the carbon–fluorine bond, but this also would add to the complexity of the definition. Thus, these restrictions were not used for PFASSTRUCTv5.

When the percentage fluorine filter is combined with the four substructures in Figure 3, then the resulting list includes approximately 15 000 structures. This number is approximately an increase of 4000 structures more than PFASSTRUCTv4. However, it is still a far more focused list and contains approximately a third as many structures as OECD2021. Of the 15 000 structures, approximately 5000 are on the list only because their percentage of fluorine formula content is 30% or more, and approximately 1300 structures are on the list only because of a substructure (Figure 3). This smaller listing demonstrates the necessity of the combined approach of substructures and percentage of fluorine.

Changing the ether substructure (Figure 2) to include other heterogenous atoms besides oxygen (Figure 3) adds approximately 100 structures that do not meet any other substructural definition. However, none of these are added to the list solely because of a substructure. They are also added to the list because of the percentage of fluorine in the molecular formula. Because new chemical substances and their associated structures are continually added to the Dashboard, the substructures may identify new chemicals in the future although, currently, they result in only limited hits.

A benefit this definition has over the more expansive OECD definition is consistency for isomers, especially with respect to structures that have a mix of halogens in the formula and attached to the structure. As discussed earlier, OECD defined PFAS as “contain at least one fully fluorinated methyl or methylene carbon atom (without any H/Cl/Br/I atom attached to it).” Further, they provide carbon tetrafluoride (DTXSID2041757) as an example of a PFAS because the carbon is fully fluorinated. However, difluoromethane (DTXSID6029597), chlorotrifluoromethane (DTXSID4052500), and bromotrifluoromethane (DTXSID5026415) are all provided as examples of compounds that are not PFAS as the carbon is not fully fluorinated with the attachment of hydrogen, chlorine, or bromine, respectively. Thus, a carbon can be fully halogenated but not fully fluorinated, and a molecule may be fully halogenated but not have any fully fluorinated carbons, so the molecule would not fit OECD2021.

**FIGURE 4:**
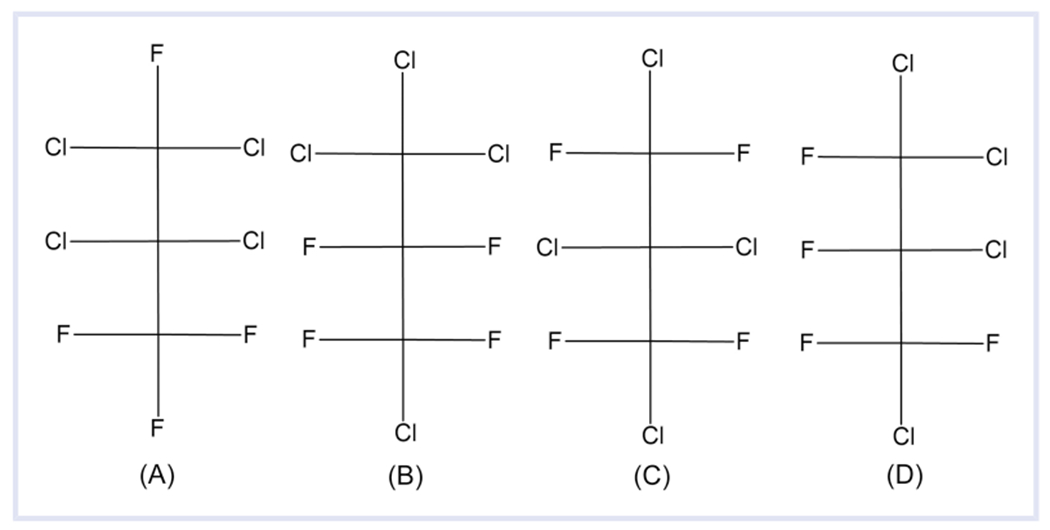
Four structures in DSSTox with the molecular formula C_3_Cl_4_F_4_: 1,1,2,2-tetrachloro-1,3,3,3-tetrafluoropropane (DTXSID10547496), 1,1,2,2-tetrafluorotetrachloropropane (DTXSID2062296), 1,2,2,3-tetrachlorotetrafluoropropane (DTXSID30865482), and 1,1,2,3-tetrachloro-1,2,3,3-tetrafluoropropane (DTXSID50547495)

With OECD’s definition, the placement of the fluorine with respect to other halogens determines whether a structure is a PFAS or not, which can lead to some perhalogenated organic compound isomers being considered PFAS, but with other isomers excluded. For example, in the Dashboard are four structures with the molecular formula C_3_Cl_4_F_4_. These structures differ *only* based on the arrangement of the chlorine and fluorine. Two of these isomers, 1,1,2,2-tetrachloro-1,3,3,3-tetrafluoropropane (DTXSID10547496; Figure 4A) and 1,1,2,2-tetrafluorotetrachloropropane (DTXSID2062296; Figure 4B) meet the OECD definition of a PFAS because they have one fully fluorinated carbon, and the other two isomers, 1,2,2,3-tetrachlorotetrafluoropropane (DTXSID30865482; Figure 4C) and 1,1,2,3-tetrachloro-1,2,3,3-tetrafluoropropane (DTXSID50547495; Figure 4D), do not. Only 1,1,2,2-tetrafluorotetrachloropropane (Figure 4B) fits PFASSTRUCTv4. With this new definition, all four isomers would be considered PFAS for PFASSTRUCTv5 as they have the same percentage of fluorine (36%). Although some may argue none of these are PFAS, at least this consideration based on the percentage of fluorine is consistent, especially when the formulas are identical. It is beyond the scope of this article to fully account for the number of fully halogenated compounds where this situation may occur with some isomers considered PFAS and some not. However, the Dashboard was also checked for C_4_Cl_5_F_5_, and six would be considered PFAS by OECD but three would not. Not all isomers are in the Dashboard.

## DISCUSSION

Numerous groups have proposed various definitions of PFAS for more than a decade, each having specific reasons. However, having numerous definitions of PFAS continues to lead to confusion. For many structures, whether or not it is a PFAS must be clarified according to the specific definition being used. PFAS have been used in numerous industries to produce a variety of commercial and consumer products (Gaines, 2022). Determining whether PFAS have been used in a particular industry or are in a particular product is not necessarily easy, but there is an additional challenge if no general agreement on the definition exists. Although different groups may focus on different PFAS subgroups for research or other efforts, having a clear and common PFAS definition allows users to understand more easily and communicate more accurately.

The concept that a set of chemicals can be simplistically defined as “per- and polyfluoroalkyl substances,” with the abbreviation of PFAS, hides the true complexity of these chemicals. A very strict interpretation of “polyfluoroalkyl substances” would include any alkyl substance with many, or a minimum of more than one fluorine attached. Poly is not normally interpreted as two, but conversely OECD2021 includes 4,4-difluoroheptane (DTXSID00509568), so some might argue 1,7-difluoroheptane (DTXSID20219354) is also a polyfluoroalkyl substance. Although all definitions thus far have placed requirements on the location of the fluorine atoms, the descriptor “polyfluoroalkyl substances” does not. PFAS are sometimes described simply as highly fluorinated organic compounds, but this also is simply too vague. This phrase does not provide a succinct definition that will not include everything with a carbon–fluorine bond but will include the desired structures. It is hard to argue that either of the heptane examples just provided are highly fluorinated. However, our approach outlined here produces yet another refined iteration to attempt to define what might encompass the PFAS class. Although this definition is also unlikely to be the ultimate, it is an attempt to focus the definition so it does not include everything with just one fluorinated carbon yet encompasses structures that previous definitions excluded but are arguably PFAS. A focused definition allows a more manageable list of chemicals (i.e., 15 000 vs. 40 000), and thus, prioritizing the compounds for research or investigation, cataloging compounds to identify properties, simply obtaining analytical standards, and so forth are easier tasks. Further, the use of formula percentage of fluorine as part of the definition allows for more flexibility as more complex structures are discovered.

With all definitions used to date, there have been several complicating issues that will cause structures to be in the gray area of what does and does not fit. One tiny change will cause a structure to no longer fit the definition despite being highly similar to others that do, such as the Figure 4 example. The inclusion of other halogens exemplifies this issue. There are many structures that perhaps would be better described as per- and polyhalogenated alkyl substances, and the placement of fluorine atoms in these halogenated structures determines whether they fit a PFAS definition. Utilizing a percentage of fluorine in the formula, PFASSTRUCTv5 may not fully eliminate the problem but should decrease the problem significantly. Generally, the concern with PFAS is that because of the strength of the carbon–fluorine bond, the fully fluorinated portion of the molecule will not degrade (USEPA, 2021c). Although the carbon–chlorine bond is weaker, to what extent substituting some fluorine atoms with chlorine will change the degradation rate is unclear. With the four isomers of C_3_Cl_4_F_4_ in Figure 4, an interesting question is whether or not the two isomers that meet the OECD definition degrade at a significantly different rate than the two isomers that do not meet the definition. If the two isomers with one fully fluorinated carbon degrade at a significantly slower rate than the two isomers that do not, then the structural definition makes more sense. Conversely, if all four isomers degrade at a similar rate, then the percentage of fluorination definition is more pragmatic.

As referenced above, OECD provides carbon tetrafluoride as an example of a PFAS because the carbon is fully fluorinated. However, difluoromethane, chlorotrifluoromethane, and bromotrifluoromethane are given as examples of non-PFAS (OECD, 2021). Arguably, they could all be described with the nonspecific phrase highly fluorinated. Although not explicitly stated, we postulate that OECD considers a fully fluorinated carbon to degrade slower than a nonfully fluorinated carbon. To investigate that idea, the predicted atmospheric lifetimes of numerous structures were compared. Using data from Forster et al. (2007), the predicted atmospheric lifetime in years, Supporting Information: Table S3, is estimated for carbon tetrafluoride (50 000 years), chlorotrifluoromethane (640 years), bromotrifluoromethane (65 years), and difluoromethane (4.9 years). This indicates a difference in the atmospheric lifetime at least for the fully fluorinated carbon, carbon tetrafluoride, but it does not indicate a quick degradation rate when at least one other halogen is bonded to the carbon, particularly chlorine. Thus, for the example of atmospheric degradation of these specific compounds, certainly, the fully fluorinated is most concerning, but concern still exists for the nonfully fluorinated carbons. Further, examination of the predicted atmospheric lifetime of other fluorinated compounds including perfluorinated compounds, hydrofluorocarbons, and halogenated compounds as examples in Supporting Information: Table S3 seems to indicate a relationship between the number of halogens, especially fluorine, rather than a relationship with the placement of the fluorine atoms (Forster et al., 2007). A similar relationship is indicated with the fluorinated ethers. CHF_2_OCF_3_ (136 years), CHF_2_OCHF_2_ (26 years), and CH_3_OCF_3_ (4.3 years) can be used for comparison due to their structural similarity. More examples would allow for more precise comparison.

Supporting Information: Table S3 notes which compounds do not meet the OECD PFAS definition. All compounds in Supporting Information: Table S3 are in PFASSTRUCTv5. If the real concern with compounds with a fully fluorinated carbon is based on slower degradation rates, then a reasonable question is: How slowly must something degrade before the degradation rate is of concern? If a PFAS definition is based on slow degradation, then the definition must specify a degradation rate. Although the predicted atmospheric lifetime of carbon tetrafluoride of 50 000 years is clearly slow, chlorotrifluoromethane is not quick at 640 years. Also, if a substance degrades relatively quickly, but it degrades to a PFAS, then the original substance is generally considered to be a PFAS.

Others have investigated PFAS degradation rates and specific bond dissociation energies, and their findings are not necessarily intuitive. Bentel and colleagues investigated the defluorination of PFAS and found that the head functional group, fluoroalkyl chain length, and position and number of C–F bonds with low bond dissociation energies affect the PFAS decay and defluorination rate. They found that perfluorocarboxylic acids defluorinated much more easily than perfluorosulfonic acids and, interestingly, fluorotelomer carboxylic acids (Bentel et al., 2019). This same group continued their investigations and found that ether oxygen atoms increase the bond dissociation energy of the C–F bonds on the adjacent –CF_2_-moieties. Further, they found that branched perfluoroalkyl ether carboxylic acids have lower percentages of defluorination (Bentel et al., 2020). This finding indicates the complexities of trying to generalize the degradation of fluorinated structures and the importance of understanding the reaction pathways. The various PFAS definitions are essentially based on defining the structures that will not degrade; therefore, understanding the complex degradation factors is critical to formulating a proper PFAS definition. Further, branching and ether groups prevent some compounds from meeting certain structural PFAS definitions; yet, Bentel’s research indicates that these same groups can decrease the defluorination reactions.

Due to a lack of data, there are few means to compare the various structures that may or may not be considered PFAS to determine effect of fluorination on degradation, toxicity, treatment, and other factors. Analysis of available bioconcentration factors (BCFs) and bioaccumulation factors (BAFs) for PFAS yields limited information. As shown in Burkhard (2021), in general, BCFs and BAFs increase with increased chain length and fluorination. Lack of information makes it difficult to compare many specific structures. However, the data in Burkhard (2021) do indicate the degree of fluorination affects BCFs and BAFs in animals. Conversely, in plants, the reverse is true. Researchers have found BAFs decrease in plants with increasing chain length (Blaine et al., 2014; Ghisi et al., 2019).

Aromatics also continue to confound the definitions of PFAS. The name PFAS implies only aliphatic carbons and excludes even perfluorinated aromatic compounds. The term PFAS is now in the popular lexicon, and there is arguably no reasonable method to introduce a new term that would include fluorinated aromatics. The new OECD definition states that there must be a fluorinated aliphatic portion. With PFASSTRUCTv5, an aliphatic portion is not required. The area is still gray, and the reasons for including or excluding aromatics can be specific to the use of the definition. In Buck et al. (2011) the P in PFAS was used as an abbreviation for either per or poly. Therefore, the A could be used similarly to do double duty as an abbreviation for either alkyl or aromatic. Hence, PFAS could be the abbreviation for per- and polyfluoro- alkyl and aromatic substances. Conversely, allowing A to mean both aliphatic and aromatic is likely to cause confusion because of the initial and continued use of the term PFAS. The aromatics can simply be referred to by a separate but similar term like per- and polyfluorinated aromatic substances (PFArS, or similar), but there would also be a gray area as to whether a structure is considered a PFAS or a PFArS. An example is given in Table 5. 2,2,2’,3,3’,4,4’,5,5,5’,6,6’-Dodecafluoro-2,5-dihydro-1,1’-biphenyl (DTXSID20525346) would be considered a PFArS, but it also fits OECD2021 because it has two fully fluorinated aliphatic carbons on the not fully aromatic ring.

Also, if the concern with PFAS is their persistence in the environment or their toxicity, then the reasoning behind a structure needing a fluorinated aliphatic carbon is not clear. Chlorinated aromatics like polychlorinated biphenyls (USEPA, 1996) and dioxins (USEPA, 2012) are highly resistant to degradation and have toxic properties, so it is likely that fluorinated aromatics would have similar properties. However, researchers have found that fluorinated aromatics degrade in the atmosphere faster than chlorinated aromatics (Brubaker & Hites, 1998; Kovacevic & Sabljic, 2017). Biodegradation of fluorinated aromatics has also been studied mainly because of their use in pesticides (Engesser & Schulte, 1989; Schreiber et al., 1980; Vargas et al., 2000). A review article found that fluorinated aromatic recalcitrance is related to the number and location of fluorine attached. Further, the authors stated “multiple fluoro-substitution substantially retards or inhibits metabolism in a much more pronounced way compared to chloro-substituted compounds” (Kiel & Engesser, 2015). Clearly, more research is needed, but fluorinated aromatics may still be a cause for concern.

A problem with all definitions based solely on the structure, including the liberal OECD definition that only requires one fully fluorinated carbon, is the degree to which structures are considered or not considered PFAS in an arbitrary way. The OECD stated that “it is key to have a coherent and consistent logic behind the PFAS definition to adequately reflect all compounds with the same structural traits, i.e., the PFAS universe” (OECD, 2021). Ironically, our analysis indicates using a purely structural definition does not allow for that. Another irony with OECD’s definition is the revised definition categorizes additional structures as PFAS because they are derivatives of compounds of PFAS. An example is that 4-((perfluorohexyl)ethyl)phenylmethanol is not purely aliphatic, but it is a derivative of 6:2 fluorotelomer iodide and thus OECD considers it a PFAS. However, polytetrafluoroethylene (PTFE) is considered a PFAS, but tetrafluoroethene, which is used to make PTFE, is not considered a PFAS by any current definition including OECD2021 (OECD, 2021). Thus, the fluorinated derivatives of PFAS are PFAS, but the fluorinated monomers that form PFAS are not necessarily PFAS. Tetrafluoroethene is included in PFASSTRUCTv5 because it is 67% fluorine. It is not an alkyl substance, but, as a monomer, it can produce many PFAS-related polymers. Currently, we are investigating PFAS-related polymers to identify PFAS-related monomers and thereby map to polymers in commerce.

The use of a percentage of fluorine as a criterion reduces the arbitrary nature of many structural definitions. However, arguably, the percentage of fluorine is still based on the structure because the molecular formula and structure are interconnected. Unlike other definitions, though, the percentage of fluorine is not substructure based. Furthermore, the percentage of fluorine may provide improved delineation of PFAS nature and shift the discussion to what percentage of fluorination is required before a structure is considered PFAS. The quantitative nature of percentage acknowledges the gray area as to whether a structure is a PFAS. Whether or not a structure has a specific substructure is a binary question and arguably does not lend itself to the complexity of PFAS structures. Conversely, the continuous function of percentage allows for a sliding scale of “PFAS-ness.” PFAS have a wide variety of properties with some PFAS of less concern than others. Arguably, the unifying concern is recalcitrance. We suggest that the definition of the percentage of fluorine is a better way to target those substances of concern. Ironically, the PFAS-ness of structures likely not only has a direct relationship with the environmental concern for a particular substance (and related structure) but also to at least some degree how useful the substance is (or was) for certain uses such as firefighting foam, mist suppressant, lubricant, cleaning agent, and so forth (Gaines, 2022).

The discussion of what is and is not a PFAS must also consider the use for the particular definition. The OECD notes “the general definition of PFASs is based on molecular structure alone and serves as a starting and reference point to guide individual users to have a comprehensive understanding of the PFAS universe and to keep the big picture of the PFAS universe in mind. At the same time, individual users may define their own working scope of PFASs for specific activities according to their specific needs by combining the general definition of PFASs with additional considerations (e.g., specific properties, use areas)” (OECD, 2021). We agree with OECD on this, but here we provide a different general definition to serve as a starting and reference point. This new definition is targeted to exclude those structures that are likely of minimal concern and allow for a more focused starting point. Thus, this new general definition may also point to a need to categorize the structures for the particular use. Hence, for a particular regulation, research project, or other use, a new PFAS definition may not be needed, but simply to classify the PFAS of focus. If the concern is simply the degradation rate, then the amount of fluorination is likely a significant factor, more so than anything else. However, if the degradation rate is the only concern, then a more scientific classification would specify the period and conditions under which the structure must degrade to not be part of the class. Classifying PFAS-based properties such as degradation or toxicity is more scientific, but the problem then lies in finding those properties for all PFAS.

Definitions of PFAS will likely continue to be specified and changed for specific regulatory and research efforts. For its proposed Drinking Water Contaminant Candidate List 5 (CCL5), the USEPA used the TSCA2021 definition to propose including PFAS as a class in the rule (USEPA, 2021a). However, based on public comments, for the final rule, the definition for CCL5 was changed to include chemicals that contain at least one of three structures: (1) R–(CF2)–CF(R’)R”, where both the CF2 and CF moieties are saturated carbons, and none of the R groups can be hydrogen; (2) R–CF2OCF2–R’, where both the CF2 moieties are saturated carbons, and none of the R groups can be hydrogen; (3) CF3C(CF3)RR’, where all the carbons are saturated, and none of the R groups can be hydrogen. The USEPA explains that the revised CCL 5 PFAS definition captures PFAS known to occur in drinking water or source water, and the definition could be revised for future cycles as more information is gathered on PFAS (USEPA, 2022). Conversely for a research effort, for an initial PFAS destruction study, Krug et al. (2022) noted that the definition of PFAS has continued to evolve, but for the purposes of their study, they considered CH_4_, CHF_3_, and C_2_F_6_ to be PFAS and used them as model PFAS compounds for a study of destruction in a research furnace. Of note, all three of these compounds are in PFASSTRUCTv5, but only CH_4_ and C_2_F_6_ are in OECD2021.

The structure’s size, including if it is a polymer or not; amount of fluorination; type and number of functional groups; amount of branching; chirality; whether it is completely aliphatic, completely aromatic, or a combination of both; and the number of other halogens present, will all likely affect the toxicity, absorption into and half-life in the body, migration in the environment, degradation rate, and other important properties. Thus, these inherent properties of a structure should be considered when either defining or focusing the categories for a respective use. Similarly, other properties may need to be considered for a respective use. For example, only nonpolymers may be of interest, or only water dissolvable PFAS may be appropriate for a research project.

The original intent of this work was not to propose a completely new definition of PFAS. The initial intent was to improve PFASSTRUCT to both simplify and increase its inclusiveness, which would improve its use as a reference. It was discovered, however, that a new way of considering what is and is not PFAS was needed. Here, we propose a general definition of PFAS that we posit to be more focused than OECD but also more inclusive than OECD. This definition can be used as a starting point for focusing or categorizing PFAS for specific uses such as management and research.

## Supplementary Material

Supplement1

## Figures and Tables

**TABLE 1:** PFASSTRUCT versions

Version	Date	Description	Number of structures
PFASSTRUCTv1	March 2018	(1) Formula must contain 4–1000 fluorine atoms; (2) structure must contain two adjacent CF_2_ groups, either in a chain or in a ring system; (3) fluorine-to-carbon ratio (#F/#C) must be ≥0.5; and (4) removal of Markush structures, charged species (e.g., anions), free radicals, and deuterium- and C^13^-labeled chemicals	4357
PFASSTRUCTv2	November 2019	R–(CF_2_)–C(F)(R’)R”, both the CF_2_ and CF moieties are saturated carbons and none of the R groups (R, R’ or R”) can be hydrogen (TSCA2021)	6648
PFASSTRUCTv3	August 2020	Structures containing one of the substructures in Figure 2	8163
PFASSTRUCTv4	November 2021	Structures containing one of the substructures in Figure 2 excluding Substructure 6	10 776

Abbreviations: CF_2_, perfluorinated methylene group; TSCA, Toxic Substances Control Act.

**TABLE 2:** DSSTox search results based on molecular formula percentage of fluorine

Percentage of fluorine	Hits including hydrogen	Hits excluding hydrogen
20	Not examined	26 000
25	Not examined	18 000
30	9800	14 000
35	Not examined	11 000
40	5800	9000

**TABLE 3:** Unique structures not included in PFASSTRUCTv4

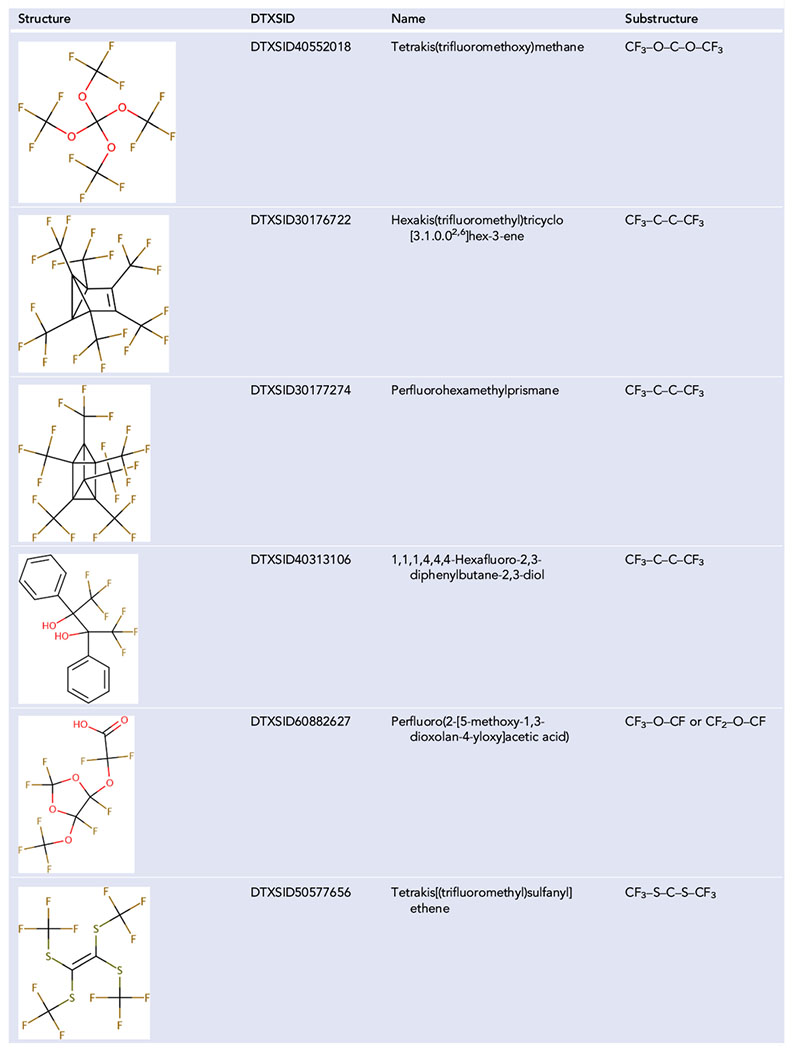
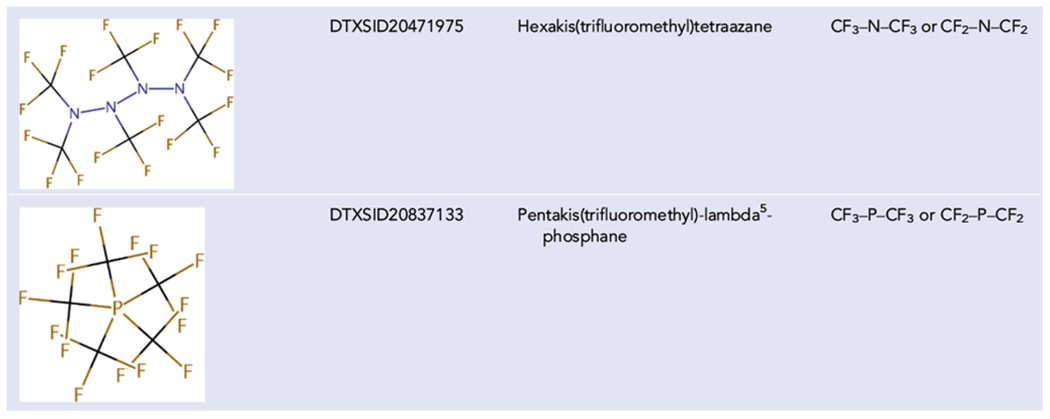

Abbreviations: CF_2_, perfluorinated methylene group; CF_3_, perfluorinated methyl group.

**TABLE 4:** Examples of structures with low fluorine percentage but would subjectively be considered PFAS

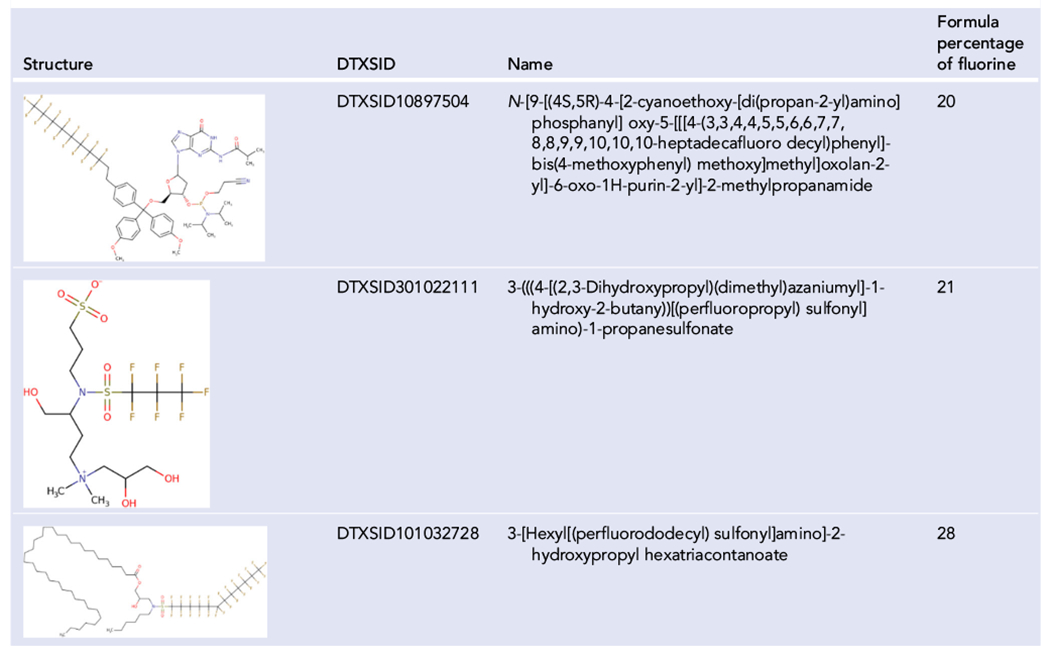

Abbreviation: PFAS, per- and polyfluoroalkyl substances.

**TABLE 5:** Structures included in PFASSTRUCTv5 that do not fit most other PFAS definitions

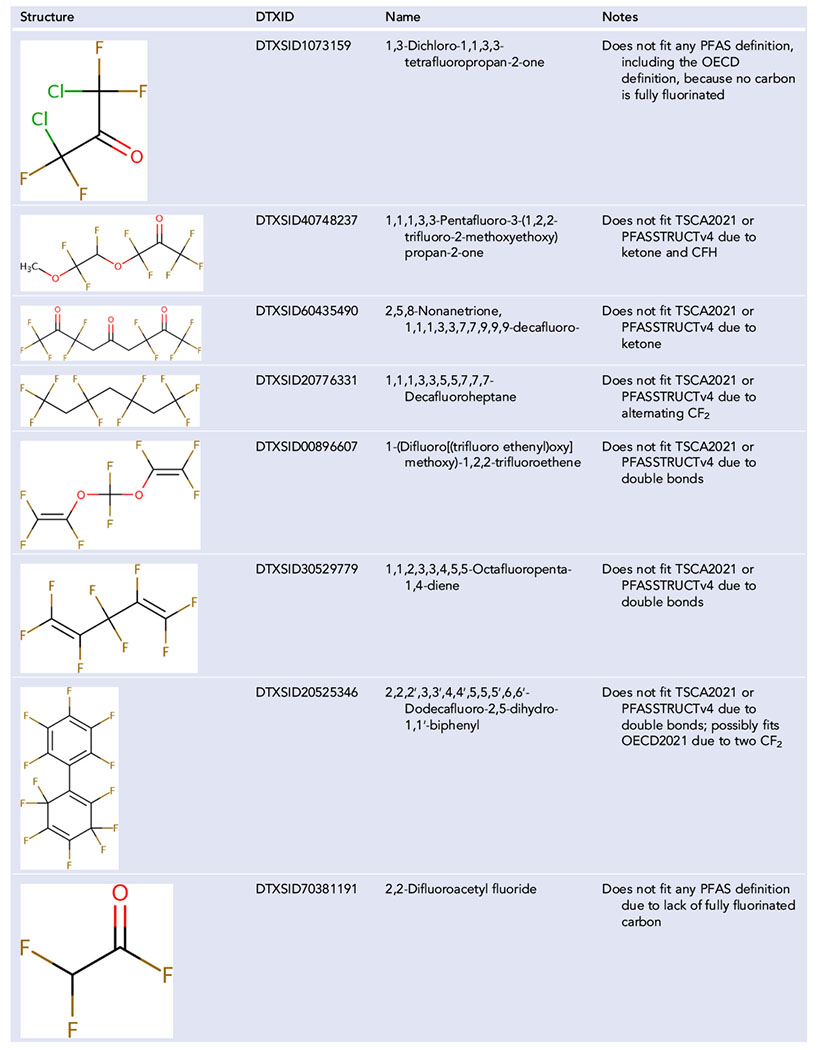
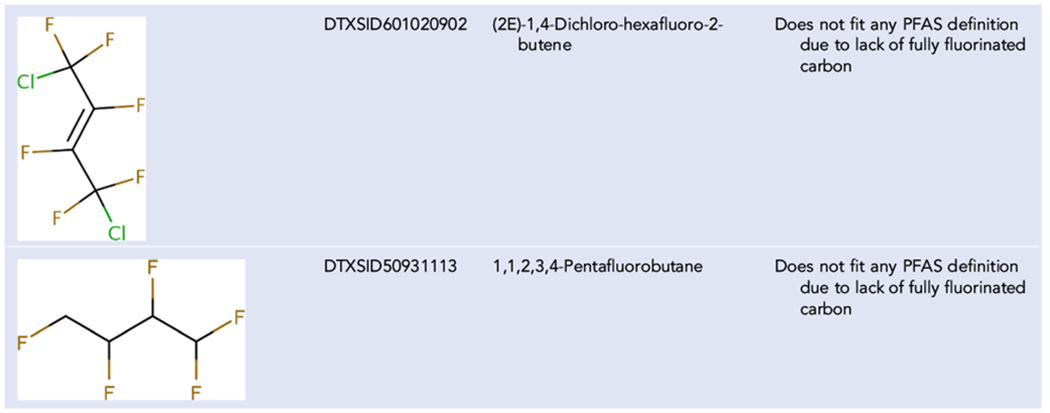

Abbreviations: CF_2_, perfluorinated methylene group; OECD, Organisation for Economic Co-operation and Development; PFAS, per- and polyfluoroalkyl substances; TSCA, Toxic Substances Control Act.

## Data Availability

All data are available online in the USEPA’s Chemistry Dashboard https://comptox.epa.gov/dashboard/.
