# Supplementary material for: A proposed approach to defining per- and polyfluoroalkyl substances (PFAS) based on molecular structure and formula
Source: Integr Environ Assess Manag. Author manuscript; Available in PMC 2024 Sep 1. (PMC10827356; doi:10.1002/ieam.4735)
Supplement: Supplement1 [file NIHMS1928343-supplement-Supplement1.docx]

**Supporting Information**

Table S1: PFAS classes from Barnabas et al.(Barnabas et al. 2022)

| **Class** | **Structural description** | **CORE structures** | **Patent structures** |
| --- | --- | --- | --- |
| A | Contains a CF_2_ group (OECD2021) | 27,058 | 1,783,651 |
| B | Contains a (AH)(AH)(F)C-C(AH)F_2_ group, where AH groups could be hydrogen or any other atom and the bond between both aliphatic carbon atoms is a single bond | 4,139 | 75,108 |
| C | Contains a (R1)(R2)(F)C-C(R3)F_2_ group, where the R groups are any atom except hydrogen and the bond between both aliphatic carbon atoms is a single bond (TSCA2021) | 3,457 | 34,197 |

Table S2: Examples of structures in the gray area regarding what percentage of fluorine is appropriate to define a PFAS structure.

| Structure | DTXSID | Name | Percent fluorine | PFAS per OECD definition? | In PFASSTRUCTv5? |
| --- | --- | --- | --- | --- | --- |
| 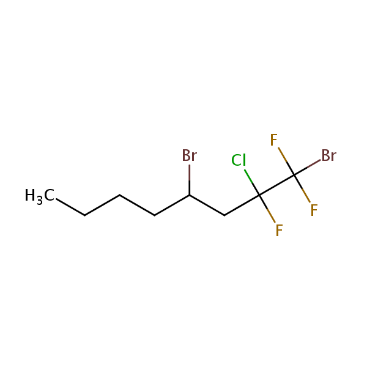 | DTXSID10382122 | 1,4-dibromo-2-chloro-1,1,2-trifluorooctane | 21 | No | No |
| 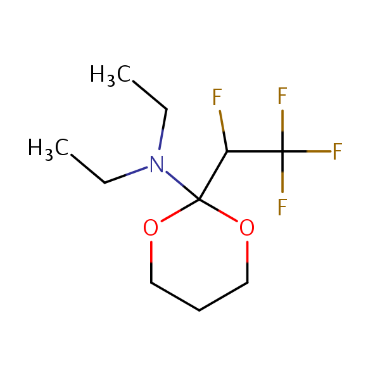 | DTXSID40519722 | N,N-Diethyl-2-(1,2,2,2-tetrafluoroethyl)-1,3-dioxan-2-amine | 24 | Yes | Yes (because of substructure) |
| 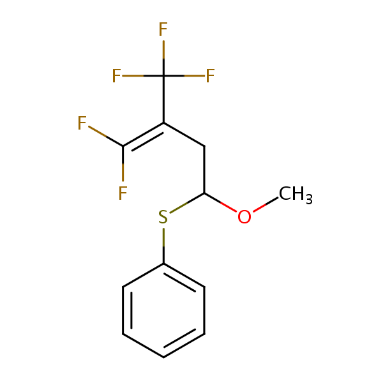 | DTXSID20809173 | ([4,4-Difluoro-1-methoxy-3-(trifluoromethyl)but-3-en-1-yl]sulfanyl)benzene | 26 | Yes | Yes (because of substructure) |
| 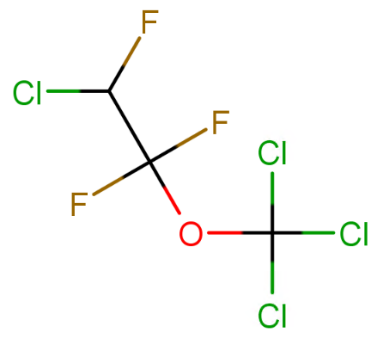 | DTXSID30963477 | 2-Chloro-1,1,2-trifluoro-1-(trichloromethoxy)ethane | 27 | Yes | No |
| 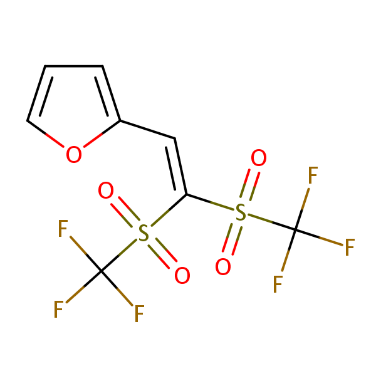 | DTXSID10711579 | 2-[2,2-Bis(trifluoromethanesulfonyl)ethenyl]furan | 29 | Yes | No |
| 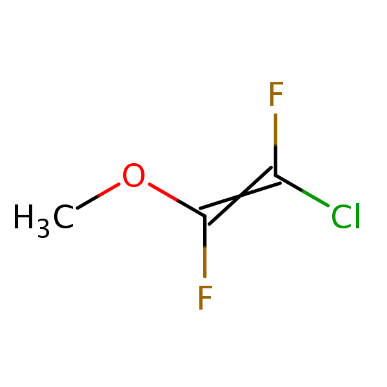 | DTXSID50777992 | 1-Chloro-1,2-difluoro-2-methoxyethene | 29 | No | No |
| 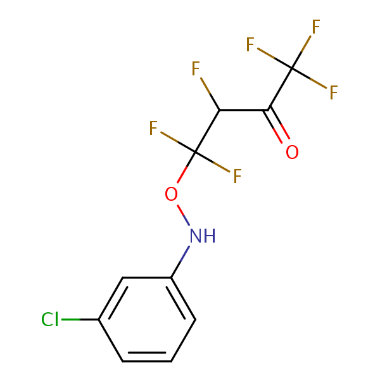 | DTXSID201023166 | 4-[(3-Chloroanilino)oxy]-1,1,1,3,4,4-hexafluorobutan-2-one | 30 | Yes | Yes |
| 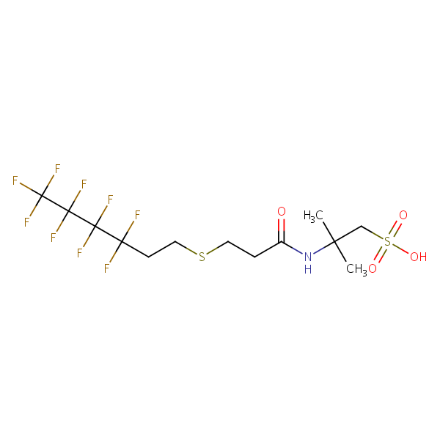 | DTXSID00892528 | 4:2 Fluorotelomer thioether amido sulfonic acid | 31 | Yes | Yes |
| 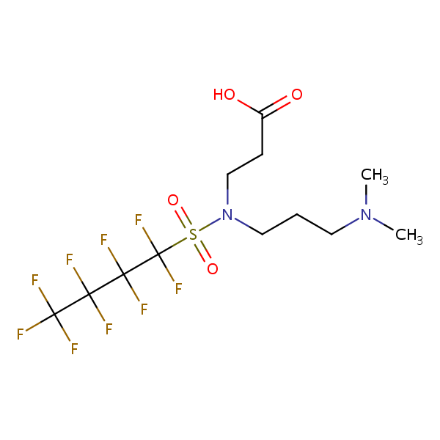 | DTXSID20882022 | N-(Perfluorobutanesulfonyl)-N-(3-dimethylaminopropyl)-3-aminopropanoic acid | 32 | Yes | Yes |
| 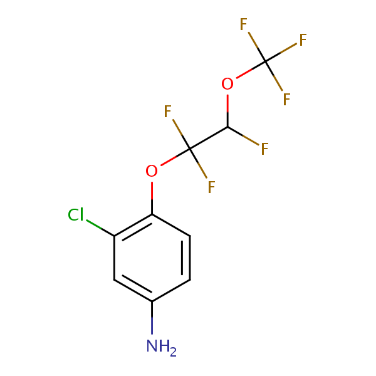 | DTXSID10576660 | 3-Chloro-4-[1,1,2-trifluoro-2-(trifluoromethoxy)ethoxy]aniline | 32 | Yes | Yes |
| 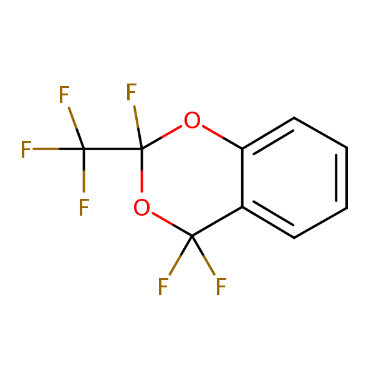 | DTXSID80661330 | 2,4,4-Trifluoro-2-(trifluoromethyl)-2H,4H-1,3-benzodioxine | 35 | Yes | Yes |
| 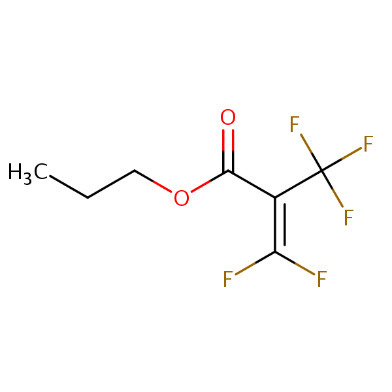 | DTXSID80804225 | Propyl 3,3-difluoro-2-(trifluoromethyl)prop-2-enoate | 36 | Yes | Yes |
| 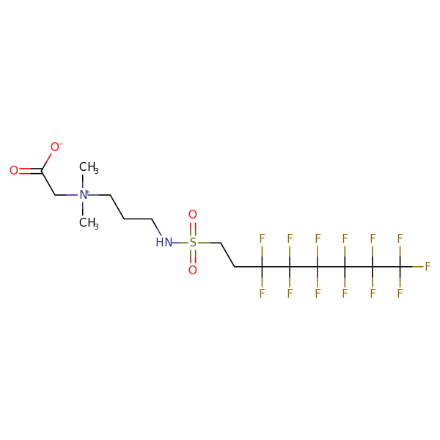 | DTXSID4041284 | 6:2 Fluorotelomer sulfonamide betaine | 37 | Yes | Yes |

**Table S3**: Predicted atmospheric lifetime of perfluorinated compounds, hydrofluorocarbons, and halogenated compounds (Forster et al. 2007)

| Type of fluorinated compound | Compound | DTXSID | Predicted atmospheric lifetime (years) | PFAS per OECD definition? |
| --- | --- | --- | --- | --- |
| Perfluorinated | CF_4_ | DTXSID2041757 | 50,000 | Yes |
| Perfluorinated | C_2_F_6_ | DTXSID2041915 | 10,000 | Yes |
| Perfluorinated | C_3_F_8_ | DTXSID9052503 | 2,600 | Yes |
| Perhalogenated | CClF_2_CF_3_ | DTXSID3026435 | 1,700 | Yes |
| Perhalogenated | CClF_3_ | DTXSID4052500 | 640 | No |
| Perhalogenated | CClF_2_CClF_2_ | DTXSID8026434 | 300 | No |
| Perhalogenated | CCl_2_F_2_ | DTXSID6020436 | 100 | No |
| Perhalogenated | CCl_2_FCClF_2_ | DTXSID6021377 | 85 | No |
| Perhalogenated | CBF_3_ | DTXSID5026415 | 65 | No |
| Fluorinated | CHF_3_ | DTXSID0026410 | 270 | No |
| Fluorinated | CH_3_CF_3_ | DTXSID9042047 | 52 | Yes |
| Fluorinated | CHF_2_CF_3_ | DTXSID1024251 | 29 | Yes |
| Fluorinated | CH_2_FCF_3_ | DTXSID1021324 | 14 | Yes |
| Fluorinated | CH_2_F_2_ | DTXSID6029597 | 4.9 | No |
| Fluorinated ether | CHF_2_OCF_3_ | DTXSID4073968 | 136 | Yes |
| Fluorinated ether | CHF_2_OCHF_2_ | DTXSID2073271 | 26 | No |
| Fluorinated ether | CH_3_OCF_2_CF_3_ | DTXSID20871517 | 5.1 | Yes |
| Fluorinated ether | CHF_2_OCH_2_CF_3_ | DTXSID5073951 | 4.9 | Yes |
| Fluorinated ether | CH_3_OCF_3_ | DTXSID5073903 | 4.3 | Yes |
| Fluorinated ether | CH_3_OCF_2_CHF_2_ | DTXSID40195282 | 2.6 | Yes |
| Fluorinated ether | CHF_2_OCHClCF_3_ | DTXSID3020752 | 2.6 | Yes |

**Figure S1:** Process used to analysis substructures of PFASSTRUCTv4

Compounds with Substructure 1

Compounds with Substructure 2

Compounds with Substructure 3

Compounds with Substructure 4

Compounds with Substructure 5

Compounds with Substructure 7

PFASSTRUCTv4

Identify which substructure lists each compound is on

Determine how many structures are unique to each substructure list

Determine if any of the substructures are extraneous or could be combined

**Figure S2:** Process used to analysis additional substructures needed to capture all PFAS

PFASSTRUCTv4

Structures with one **CF2**

TSCA2021

Full analysis list of candidate PFAS

Remove structures with less than 3 fluorine atoms

Analyze SMILES, remove structures with only 3 fluorine atoms contained in one CF3 group

Remove structures with only 6 fluorine atoms contained in two CF3 groups, where those two CF3 groups are not connected to same carbon to create narrowed analysis list

Remove structures on PFASSTRUCTv4

Determine new substructures needed to capture PFAS

**Figure S3:** Process used to analysis structures resulting from percent fluorine searches

Structures with 20% fluorine

Structures with 25% fluorine

Structures with 30% fluorine

Full analysis list of candidate PFAS based on percent fluorine

Compared to narrowed analysis list from substructure analysis

Determine most appropriate percent fluorine to use to capture PFAS

Determine if a combination of percent fluorine and substructure-based criteria most efficiently capture PFAS

Structures with 35% fluorine

Structures with 40% fluorine
